# Catheter-related septic thrombophlebitis of the superior vena cava involving the atrial septum: a case report

**DOI:** 10.1186/1757-1626-1-272

**Published:** 2008-10-24

**Authors:** Stavros Tzortzis, Stavros Apostolakis, Konstantinos Xenakis, Georgios Spiropoulos, Kyriakos Lazaridis

**Affiliations:** 1 Department of Cardiology, Army Veterans Hospital, Athens, Greece; 2 Department of Clinical Echocardiography, Army Veterans Hospital, Athens, Greece

## Abstract

**Background:**

Intravascular catheters provide necessary vascular access, for intravenous therapy, blood sampling and pressure monitoring. However, their use is often associated with serious local and systemic complications including local site infection, intravascular catheter-related bloodstream infections, septic thrombophlebitis, and endocarditis.

**Case presentation:**

We present a case of a 72 year old postoperative patient presented with persistent fever. Transthoracic and transesophageal echocardiograms demonstrated a lesion in the superior vena cava, protruding into the right atrium and infiltrating the atrial septum. Candida albicans grew in blood cultures as well as in the subclavian catheter tip culture. Anti-fungal and antithrombotic therapy was initiated. After 2 weeks treatment the lesion was diminished.

**Conclusion:**

Transthoracic and transesophageal echocardiography has been proved efficient and cost-effective in guiding therapy in cases of catheter related infections. In the presented case the lesions in vena cava and the involvement of the endocardium were early identified by echocardiography. Moreover, a follow-up echocardiogram confirmed the efficiency of the therapeutic approach.

## Background

Intravascular catheters provide necessary vascular access, for intravenous therapy, blood sampling and pressure monitoring. However, their use is associated with serious local and systemic complications, including local site infection, intravascular catheter-related bloodstream infections, septic thrombophlebitis, and endocarditis. The majority of serious catheter-related infections are associated with central venous catheters. Implementation of evidence-based preventive tactics is pivotal in reducing the risk for serious catheter-related infection [1].

## Case presentation

A 72 year old Caucasian woman was referred to our department after a non-ST elevation myocardial infarction (NSTEMI). The patient was previously attended in the surgery department of her regional General Hospital where she was subjected to enterectomy due to postoperative ileus. She was a non-smoker, she had normal body mass index (BMI) and no personal or family history of cardiovascular disease was reported to us.

During surgery a subclavian central venous catheter had been placed to substantiated uninterrupted fluid infusion and central venous pressure (CVP) monitoring. Post-surgery she developed fever. Cultures were obtained from the surgical trauma, peripheral veins and the subclavian central venous catheter. Empirical antibiotic treatment was initiated. However, while still febrile the patient had to be transferred to our department after an episode of chest pain and laboratory findings indicative of a NSTEMI. On admission to our department the patient was febrile, mildly tachycardic with normal blood pressure and normal respiration rate. She did not report dyspnea, chest discomfort or abdominal pain and her clinical examination was relatively normal. Initial electrocardiogram showed sinus tachycardia and non specific T wave abnormalities at anterior leads. The chest radiography revealed a normal cardiac silhouette and a small right pleural effusion. Laboratory tests yielded normal white cell count, mild normochromic normocytic anemia (hemoglobin: 10.3 g/dL, mean corpuscular volume: 89.7 fL) and increased levels of acute-phase proteins (C-reactive protein: 12.7 mg/dL, erythrocyte sedimentation rate: 55 mm/hr). The cultures obtained from the surgical trauma turned out negative, while Candida albicans was isolated from both the subclavian venous catheter and the blood cultures.

**Figure 1:**
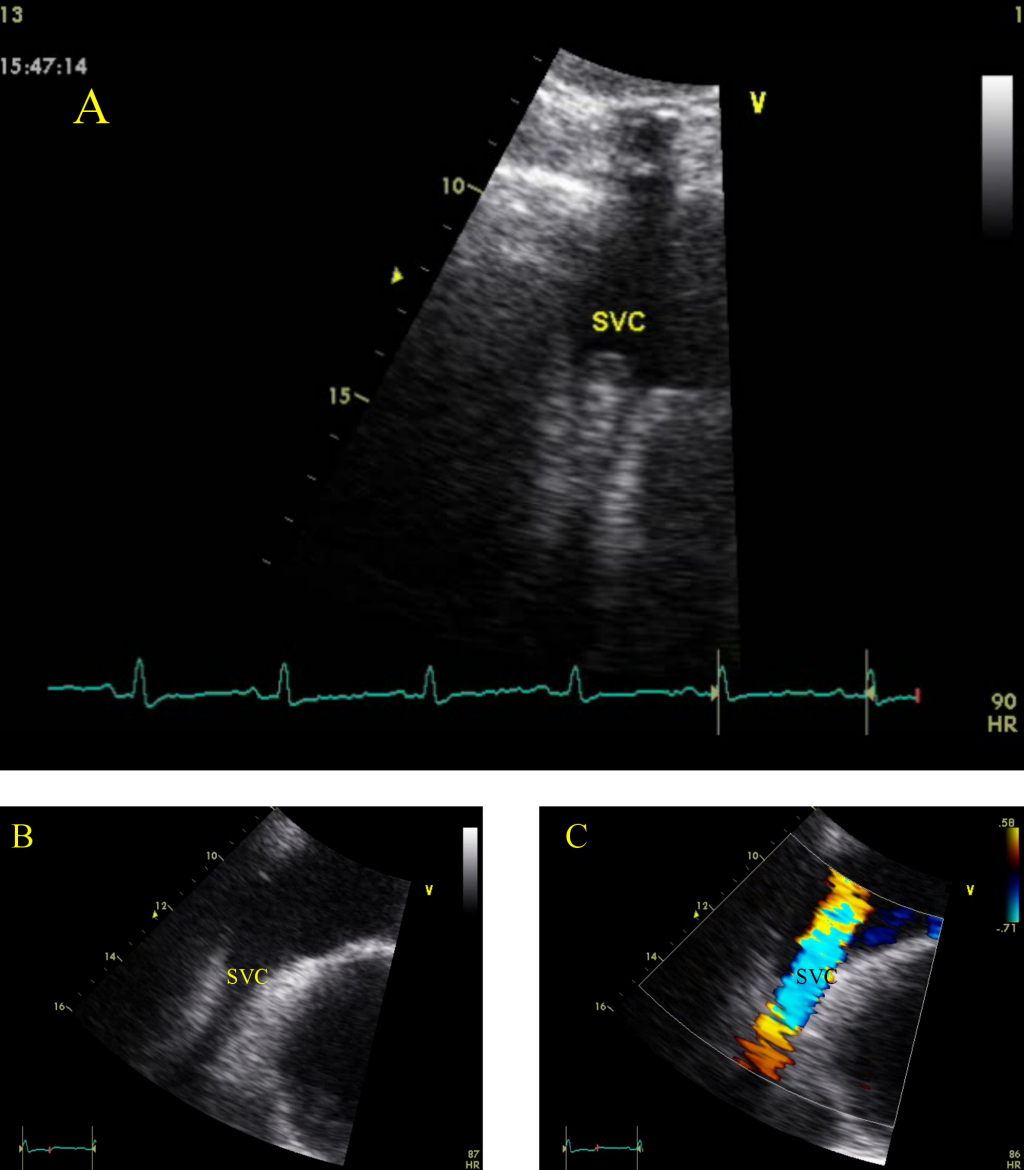
**Transthoracic echocardiographic imaging of right atrium and superior vena cava (subcostal view).** A round solitary lesion, protruding into the right atrium is clearly visualized (A). The lesion has been diminished 15 days after anti-fungal and anti-thrombotic treatment (B, C).

**Figure 2:**
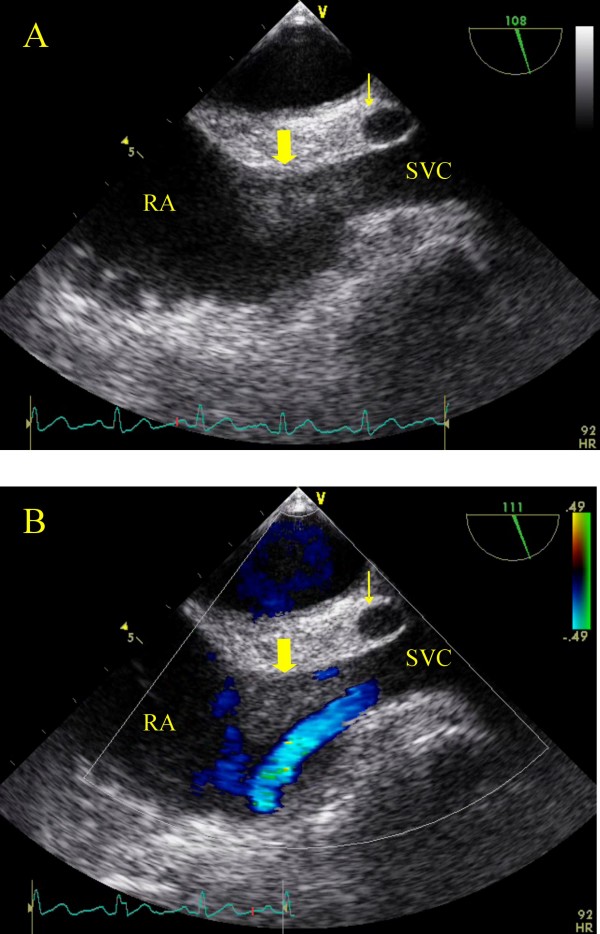
**Transesophageal echocardiographic imaging-bicaval view-confirmed a 2.5 × 1.5 round solitary lesion with distinctive borders attached in the SVC, protruding into the right atrium (Thick arrow).** The echo lucent area-marked with the thin arrow-most probably presents an abscess of the interatrial septum (A, B). Flow from the superior vena cava was obstructed (B).

**Figure 3:**
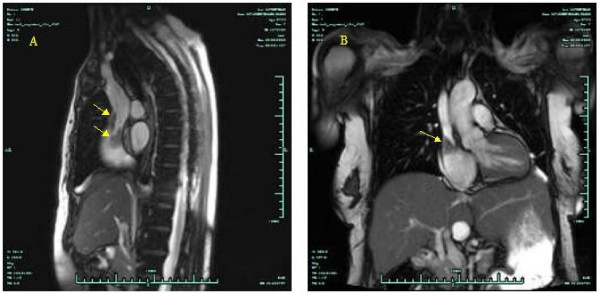
Magnetic resonance imaging (MRI) demonstrating a solitary mass attached in superior vena cava-atrial junction.

The patient was subjected to a transthoracic ultrasound where a mass was noted in the right atrium originating from the superior vena cava (SVC). Flow from the SVC was obstructed (Fig. 1, Panel A). A transesophageal echocardiogram was also performed and revealed a 2.5 × 1.5 round solitary lesion with distinctive borders attached in the SVC, protruding into the right atrium (Fig. 2, Panels A, B). An echo lucent, flow-free cavity in the atrial septum was also noted suggestive of an atrial septal abscess (Additional file 1). No valve vegetations were demonstrated. The lesion was also confirmed by magnetic resonance imaging (MRI) (Fig. 3).

Based on microbiology and echocardiographic findings, the patient was put-on low molecular weight heparin (LMWH) and amphotericin-B. After 6 days treatment, the fever resolved, and 15 days later a repeated transthoracic echocardiogram demonstrated a barely noticed thickening of the SVC wall (Fig. 1, Panel B, C). The atrial septum was intact. The patient was discharged on LMWH and no adverse events were recorded in the next six-month follow-up.

## Discussion

Septic thrombophlebitis is a life-threatening condition associated with use of central venous devices [2,3]. Sole use of antimicrobials is rarely effective and therapeutic strategies should also include catheter removal and anticoagulation [4]. In certain instances thrombolysis, thrombectomy or venotomy have been applied. The latter approach is technically impossible for great central veins, although surgical thrombectomy has been successfully performed [5,6] and medical lysis of the thrombus is feasible [7-9].

Candida spp. has been reported as the leading offending agent of fungal hospital-acquired bloodstream infections, being responsible for 8% of the reported cases [1,10,11]. A problem that often arises when dealing with Candida related septic thrombophlebitis is the increasing resistance of Candida spp. to commonly used antifungal agents. It has been reported that 10% of Candida albicans bloodstream isolates from hospitalized patients were resistant to fluconazole [1] and 48% of Candida bloodstream infections were caused by non-albicans species, which are more likely to demonstrate resistance to conventional antifungal agents [1]. It was therefore essential to take into account the possibility of fluconazole resistant when we considered the therapeutic options in the presented case of Candida related septic thrombophlebitis.

Finally, echocardiography is a non-invasive and inexpensive test that can be easily applied in any clinical setting. The use of echocardiography has been proved efficient and cost-effective in guiding therapy in cases of catheter related infections [12]. Lesions in vena cavas and involvement of the endocardium can be early identified and influence therapeutic decisions such as duration of antibiotic therapy and the need to proceed to surgery. A follow up echocardiogram can also confirm the efficiency of the therapeutic approach. However, distinguishing catheter related septic thrombophlebitis from catheter related thrombosis is virtually impossible on echocardiographic grounds alone and invariably requires clinical correlation.

## Conclusion

In conclusion, we presented the case of a woman with catheter related thrombophlebitis successfully treated with LMWH and amphotericin-B. Transthoracic and transesophageal echocardiography were proved valuable tools in establishing the diagnosis of vena cava thrombophlebitis and assessing the response to anti-fungal treatment.

## Abbreviations

NSTEMI: non-ST elevation myocardial infarction; BMI: body mass index; CVP: central venous pressure; SVC: superior vena cava; MRI: magnetic resonance imaging; LMWH: low molecular weight heparin.

## Consent

Written informed consent was obtained from the patient for publication of this case report and accompanying images. A copy of the written consent is available for review by the Editor-in-Chief of this journal.

## Competing interests

The authors declare that they have no competing interests.

## Authors' contributions

ST and SA wrote the manuscript and edited the images. KX performed and interpreted the echocardiographic images. GS and KL suggested the presentation of this case and reviewed the manuscript. All authors read and approved the manuscript.

## Supplementary Material

Additional file 1 Transesophageal echocardiographic imaging of right atrium and vena cavas-bicaval view-revealed a 2.5 × 1.5 round solitary lesion with distinctive borders attached in the SVC, protruding into the right atrium. The echo lucent area in the interatrial septum presents most probably an abscess. Color doppler did not detect flow in the cavity. Flow from the superior vena cava was obstructed. Click here for file
